# CD44 standard isoform is involved in maintenance of cancer stem cells of a hepatocellular carcinoma cell line

**DOI:** 10.1002/cam4.1968

**Published:** 2019-01-12

**Authors:** Ryoma Asai, Hiroyuki Tsuchiya, Masataka Amisaki, Kazuki Makimoto, Ai Takenaga, Tomohiko Sakabe, Shotaro Hoi, Shigemi Koyama, Goshi Shiota

**Affiliations:** ^1^ Division of Molecular and Genetic Medicine, Graduate School of Medicine Tottori University Yonago Japan; ^2^ Faculty of Medicine, Division of Surgical Oncology, Department of Surgery Tottori University Yonago Japan; ^3^ Faculty of Medicine, Division of Organ Pathology, Department of Pathology Tottori University Yonago Japan

**Keywords:** cancer stem cell, CD44, hepatocellular carcinoma, NOTCH3, oxidative stress

## Abstract

Hepatocellular carcinoma (HCC) is one of the leading causes of cancer death worldwide. Cancer stem cells (CSCs) have attracted attention as a novel therapeutic target for cancer because they play important roles in the development and aggravation of cancer. CD44 is expressed as a standard isoform (CD44s) and several variant isoforms. CD44v is a major isoform expressed on CSCs of a variety of tumors and has been extensively studied. However, HCC tissues dominantly express CD44s, whose function in CSCs remains unclear. In the present study, we investigated the roles of CD44s in CSCs of HCC. Knock‐out of the CD44 gene in HuH7 HCC cells on which only CD44s is expressed resulted in decreased spheroid formation and increased drug sensitivity. The expression of CSC marker genes, including CD133 and EpCAM, was significantly downregulated in the spheroids of CD44‐deficient cells compared with those in the spheroids of HuH7 cells. In addition, CD44 deficiency impaired antioxidant capacity, concomitant with downregulation of glutathione peroxidase 1 (GPX1) and thioredoxin. Because GPX1 uses the reduced form of glutathione (GSH) to regenerate oxidized cellular components, GSH levels were significantly increased in the CD44‐deficient cells. We also found that NOTCH3 and its target genes were downregulated in the spheroids of CD44‐deficient cells. NOTCH3 expression in HCC tissues was significantly increased compared with that in adjacent nontumor liver tissues and was correlated with CD44 expression. These results suggest that CD44s is involved in maintenance of CSCs in a HCC cell line, possibly through the NOTCH3 signaling pathway.

## 1 INTRODUCTION

Primary liver cancer accounts for more than 30 000 deaths each year in Japan and is one of the leading causes of cancer death worldwide.^1^ Hepatocellular carcinoma (HCC) is the most prevalent subtype of primary liver cancer.^1^ Despite significant advances in HCC treatment, the recurrence rate is still high at 60%‐70%.^2^, ^3^ Thus, increasing attention is being focused on the mechanisms by which cancer cells acquire the malignant phenotype.

Accumulating evidence suggests that recurrence and metastasis in many cancers can be attributed to cancer stem cells (CSCs).^4^, ^5^ Because CSCs have the ability of self‐renewal and differentiation, CSCs are indispensable for initiating and maintaining tumor phenotypes while other cancer cells (non‐CSCs) do not have such properties.^6^ The existence of CSCs was first demonstrated in acute myeloid leukemia.^7^ Since then, using specific CSC markers, the existence of CSCs has been proven in various tumors such as brain, breast, lung, colon, and liver.^8^, ^9^, ^10^, ^11^, ^12^ Several studies reported that liver CSCs express several markers such as epithelial adhesion molecule (EpCAM), CD13, CD44, and/or CD133 and that the expression of these molecules on HCC cells is correlated with poor prognosis.^12^, ^13^, ^14^ Because CSCs display the features of tumorigenicity and resistance to conventional chemotherapy and radiotherapy, it is suggested that surviving CSCs eventually cause tumor recurrence and metastasis although conventional therapies could eliminate non‐CSCs.^4^, ^5^ Therefore, the removal of CSCs is important to cure cancer completely.

A transmembrane glycoprotein CD44 is the most frequently observed CSC marker.^15^ The gene encoding human CD44 consists of 20 exons, 10 of which are variant exons, and produces several isoforms.^16^ The CD44 variant isoform containing the variant exons 8 and 10 (CD44v) is a major CSC marker, and frequently upregulated on CSCs of many tumors such as gastric, colorectal, breast, and prostate cancers.^17^ Because this isoform promotes glutathione (GSH) synthesis by interacting with xCT, a glutamate‐cystine transporter, CD44v enhances antioxidant capacity in CSCs.^18^ In contrast, the CD44 standard isoform (CD44s) lacking variant exons is found in various cells such as mesenchymal stromal and hematopoietic cells.^19^ However, it has been reported that hepatic CSCs dominantly express CD44s and that CD44s is involved in epithelial mesenchymal transition (EMT).^20^, ^21^, ^22^ We have previously reported that CD44 expression in HCC tissues, which is assessed by an antibody against total CD44, was significantly associated with poor prognosis while no significant association was observed between the prognosis and CD44v expression, which is assessed by a CD44v‐specific antibody.^23^ These results prompted us to investigate the biological functions of CD44s in controlling antioxidant capacity and CSCs in HCC.

Genome editing technologies are widely used for investigating molecular functions of gene products.^24^ Clustered regularly interspaced short palindromic repeats/CRISPR‐associated proteins 9 (CRISPR/Cas9) system is one of the most established genome editing technologies derived from prokaryotes, where this system plays a role as an immune system against phages and plasmids.^25^, ^26^ Cas9 is an endonuclease and cleaves a specific DNA sequence indicated by a guide RNA at a position 3‐bp upstream of the trinucleotide (5′‐NGG‐3′) protospacer‐adjacent motif (PAM).^24^ Mutation(s) is thought to be induced during a DNA repair process known as nonhomologous end joining.^24^ The CRISPR/Cas9 system has proven its applicability for cancer research.^27^ This prompted us to study the function of CD44s in HCC cells.

In this study, we established CD44‐knocked out (CD44‐KO) cells from human HuH7 HCC cells, in which only CD44s is expressed, by means of the CRISPR/Cas9 system, and investigated the phenotypic changes in CSC properties of CD44‐KO cells.

## 2 MATERIALS AND METHODS

### 2.1 Knocking out the CD44 gene in HuH7 cells

Human hepatocellular carcinoma HuH7, HLE, HLF, HuH6, PLC/PRF/5, and HepG2 were purchased from the JCRB cell bank (Osaka, Japan) and was maintained in DMEM (Nissui Pharmaceutical; Tokyo, Japan) supplemented with 10% inactivated FBS (Sigma‐Aldrich; St. Louis, MO, USA).

Single‐guide RNA (sgRNA) targeting the CD44 gene was designed by GeneArt CRISPR Search and Design Tool (https://apps.thermofisher.com/apps/crispr/index.html), crispr grna design tool (https://www.atum.bio/eCommerce/cas9/input) and CRISPRdirect (https://crispr.dbcls.jp/). Off‐target sequence was searched by using GGGenome (https://gggenome.dbcls.jp/en/). The sequence CTACAGCATCTCTCGGACGGAGG (underline indicates PAM sequence) commonly provided by all three programs was used as the sgRNA. A double‐stranded oligo DNA harboring the sequence (Table S1) was ligated into pSpCas9(BB)‐2A‐GFP (PX458) (Addgene, #48138; Cambridge, MA, USA), which expresses both sgRNA and SpCas9.^24^ The plasmid DNA was transfected into HuH7 cells with Viofectin (Viogene, New Taipei City, Taiwan). It is of note that the cells used for the transfection was not sorted and thereby included both CD44‐positive and negative cells. Sequencing analysis of the genomic DNA of independent clones obtained by limited dilution was performed by BigDye Terminator v3.1 Cycle Sequencing Kit (Thermo Fisher Scientific; Waltham, MA, USA). The HuH7 HCC cells in which the CD44 gene is knocked out were designated CD44‐KO cells.

### 2.2 Spheroid formation assay

HuH7 or CD44‐KO cells were plated in a ultra‐low attachment 24‐well plate (Corning; Corning, NY, USA) at 3000 cells/well and were cultured for 7 days in a spheroid culture medium, which was prepared by adding 20 ng/mL of recombinant human epidermal growth factor (PeproTech; Rocky Hill, NJ, USA), 20 ng/mL of recombinant human basic fibroblast growth factor (PeproTech), 1 × B27 (Thermo Fisher Scientific), and 0.52% methylcellulose (Sigma‐Aldrich), to Ham's F‐12 medium (Nacalai Tesque; Kyoto, Japan). The number of spheroids was counted by ImageJ software (Bethesda, MD, USA). NOTCH3‐specific siRNAs (siN3‐1 and siN3‐2) (Silencer Select siRNA s9640 and s532202, respectively, Thermo Fisher Scientific) and negative control siRNA (siNC) (Silencer Select Negative Control No. 2 siRNA, Thermo Fisher Scientific) were transfected with Lipofectamine RNAiMax (Thermo Fisher Scientific) into HuH7 cells one day before the start of spheroid culture.

### 2.3 Reverse transcription quantitative PCR (RT‐qPCR)

Cells in monolayer culture were prepared by culturing HuH7 or CD44‐KO cells at 6.5 × 10^4 ^cells/well in a 12‐well cell culture plate (Violamo; Osaka, Japan) for 4 days. Spheroids were prepared by plating cells at 5.0 × 10^4 ^cells/well in the ultra‐low attachment 24‐well plate, and culturing the cells in the spheroid culture medium for 7 days. Total RNA was recovered by TRIzol (Thermo Fisher Scientific). Following treatment with DNase (Nippon Gene; Tokyo, Japan), complementary DNA was synthesized by Super Script First‐Strand Synthesis for RT‐PCR Kit (Thermo Fisher Scientific). QPCR was performed by THUNDERBIRD SYBR qPCR Mix (Toyobo, Otsu, Japan) using ViiA 7 Real‐time PCR System (Thermo Fisher Scientific). Primers used in the present study were summarized in Table S1. Relative mRNA expression levels were calculated by using β‐actin as an internal control. For comparison of monolayer culture and spheroids, HPRT1 was used as an internal control instead of β‐actin, whose expression level was readily affected by the culture conditions.

### 2.4 Western blot analysis

Cells in monolayer culture were incubated in a 6‐cm dish (Violamo) at 5.0 × 10^5 ^cells/dish for 4 days. Spheroids were prepared by seeding cells to a Sumilon PrimeSurface 90‐mm dish (Sumitomo Bakelite; Tokyo, Japan) at 1.0 × 10^6 ^cells/dish and incubated in spheroid culture medium for 7 days. The cells were washed three times with phosphate‐buffered saline and were lysed in 150 µL RIPA buffer (50 mmol/L Tris‐HCl (pH 7.9), 150 mmol/L NaCl, 1% NP‐40, 0.5% sodium deoxycholate, 0.1% sodium dodecyl sulfate (SDS)). After freezing the lysate at −80°C, the protein concentration was determined by the Bradford reagent (Bio‐Rad Laboratories; Hercules, CA, USA) and was adjusted with the RIPA buffer and 5 × sample buffer (60 mmol/L Tris‐HCl (pH 7.9), 14.4% β‐mercaptoethanol, 2% SDS, 25% glycerol). SDS‐polyacrylamide gel electrophoresis and immunoblotting were performed by standard procedures. Antibodies against actin (sc‐1616; Santa Cruz Biotechnology; Dallas, TX, USA), CD44 (#3570; Cell Signaling Technology; Danvers, MA, USA), glutathione peroxidase 1 (GPX1) (ab22604; Abcam; Cambridge, UK), glyceraldehyde‐3‐phosphate dehydrogenase (GAPDH) (sc‐365062; Santa Cruz Biotechnology), NOTCH1‐3 (#3608, #5732, and #5276, respectively; Cell Signaling Technology), superoxide dismutase 1 (SOD1) (sc‐11407; Santa Cruz Biotechnology), and thioredoxin (TXN) (M013‐3; MBL; Nagoya, Japan) were used.

### 2.5 Determination of cellular reactive oxygen species

Cellular reactive oxygen species were measured by using DCFDA‐Cellular Reactive Oxygen Species Detection Assay Kit (Abcam) with slight modifications. Cells were seeded to a dark 96‐well microplate (Thermo Fisher Scientific) at 1 × 10^4 ^cells/well and were allowed to attach overnight. The cells were washed twice with 1 × buffer solution and were incubated with 100 µL of DCFDA solution for 45 minutes at 37°C. After being washed twice with 1 × buffer solution, the cells were exposed to 0, 50, or 100 µmol/L of hydrogen peroxide (H_2_O_2_) (Nacalai Tesque) or *tert*‐butyl hydroperoxide (TBHP) (Abcam) for 5 hours at 37°C. Fluorescence intensity (excitation, 485 nm; emission, 535 nm) was measured by a plate reader (Tecan; Männedorf, Switzerland).

### 2.6 Determination of cellular GSH content

GSH and GSSG (oxidized glutathione) in cells at a confluency of approximately 80% on a 10‐cm dish were determined by GSH/GSSH Quantification Kit (Dojindo; Kumamoto, Japan) according to the manufacturer's protocol. Absorbance at 415 nm was measured by using the plate reader.

### 2.7 Cell viability assay

Cells were seeded to a 96‐well plate (Violamo) at 5.0 × 10^3 ^cells/well and were allowed to attach overnight. The cells were treated with 0‐20 µmol/L of sorafenib (Bayer; Leverkusen, Germany), or 0‐50 µg/mL of 5‐fluorouracil (5‐FU) (Nacalai Tesque) for 72 hours. The concentration of solvent (dimethyl sulfoxide) was kept at 0.1%. Cell counting kit‐8 (Dojindo) was used to determine cell viability according to the manufacturer's protocol. Absorbance at 450 and 600 nm was measured by using the plate reader.

### 2.8 Analysis of human samples

Liver specimens from 92 HCC patients (Table 1) were obtained at Tottori University Hospital between 2004 and 2013 and immediately stored in RNAlater (Qiagen; Valencia, CA, USA) at −80°C. RNA from frozen specimens was recovered and purified using TRIzol reagent and RNeasy Plus Kit (Qiagen), according to the manufacturer's instructions. CDNA synthesis and quantitative RT‐PCR were performed as described above. The clinical data of the patients were collected from medical records. Medical records were reviewed retrospectively after approval by the Institutional Review Board of our institution in accordance with the ethical standards laid down in the 1964 Declaration of Helsinki and its later amendments (Institutional Review Board approval number: 18A071).

**Table 1:** Clinical and demographic characteristics of hepatocellular carcinoma patients

Number of patients	92
Age (y)	67.2 ± 10.7^a^
Gender (male/female)	79/13
Etiology (HBV/HCV/nonB·nonC)	40/23/29
Child‐Pugh score (5/6/≥7)	67/19/6
Number of tumors (1/≥2)	80/13
Tumor size (cm)	5.3 ± 4.1^a^
vp (0/1/2/3/NA)	64/19/4/2/3
TNM stage (IA/IB/II/IIIA/IIIB/IVA)	8/30/38/7/7/2
Fibrosis stage (HAI‐IV^b^) (≤3/4/NA)	61/27/4
Survival period (y)	4.2 [2.8‐7.3]^c^

a Mean ± standard deviation.

b Histological activity index IV (Ishak K, et al J Hepatol. 1995;22:696‐699).

c Median [interquartile range].

### 2.9 Statistical analysis

Independent samples, of which numbers are over 3, were analyzed, and all experimental values were expressed as mean ±standard deviation (SD). The differences between the two groups were assessed by Student's *t* test. *P* value <0.05 was considered as statistically significant.

## 3 RESULTS

### 3.1 Knocking out the CD44 gene in HuH7 cells

**Figure 1:**
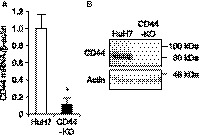
CD44 expression in HuH7 and CD44‐KO cells. A, relative mRNA expression levels of CD44 to β‐actin. *P < 0.05, vs HuH7 cells; Student's *t* test. B, CD44 protein expression. Actin is shown for loading control

Since HuH7 cells express only CD44s isoform,^22^ we knocked out the CD44 gene in HuH7 cells by employing the CRISPR/Cas9 system to clarify whether CD44s plays a role in HCC. Several clones were obtained by limited dilution of HuH7 cells transfected with plasmid DNA expressing SpCas9 and sgRNA targeting the CD44 exon 2, one of which showed 8‐bp deletion and 256‐bp insertion within each CD44 allele (Figure S1). This clone also showed remarkable decreases in expression of mRNA and protein of CD44 (Figure 1A,B). No mutation was found in the inhibitor of nuclear factor kappa B kinase subunit gamma (IKBKG) gene, which is the only one gene that was predicted to contain a potential off‐target site by searching with 12‐mer plus PAM sequence (Figure S2). We hereafter used this clone as CD44‐KO cells.

### 3.2 Lowering of antioxidant capacity in CD44‐KO cells

**Figure 2:**
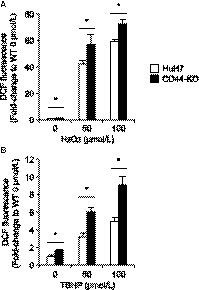
Cellular oxidative stress. A, oxidative stress in cells treated with 0‐100 µmol/L of H_2_O_2_ for 5 h. B, oxidative stress in cells treated with 0‐100 µmol/L of TBHP for 5 h. *Open bars*, HuH7 cells; *filled bars*, CD44‐KO cells. *P < 0.05; Student's *t* test

CD44v was reported to increase cellular antioxidant capacity by activating the biosynthesis of GSH.^18^ High antioxidant capacity is a common feature of CSCs,^18^, ^28^, ^29^ prompting us to investigate the effect of CD44s deficiency on cellular antioxidant capacity. CD44‐KO cells treated with H_2_O_2_ or TBHP showed significantly higher oxidative stress than HuH7 cells at 0, 50, and 100 µmol/L of their concentrations (Figure 2A,B), suggesting that CD44s increases cellular antioxidant capacity in HuH7 cells.

### 3.3 Decreased expression of antioxidant factors in CD44‐KO cells

**Figure 3:**
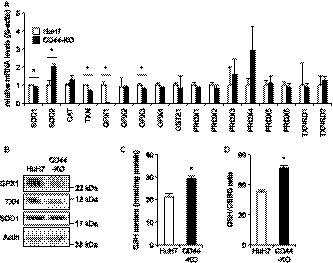
Expression of antioxidant factors. A, relative mRNA expression levels of antioxidant factors to β‐actin. B, protein expression levels of GPX1, TXN, and SOD1. Actin is shown for loading control. C, cellular GSH content. D, cellular GSH to GSSG ratio. *Open bars*, HuH7 cells; *filled bars*, CD44‐KO cells. *P < 0.05, vs HuH7 cells; Student's *t* test

The mRNA expression of antioxidant factors was determined by RT‐qPCR. In CD44‐KO cells, SOD1, TXN, GPX1, and GPX3 were significantly downregulated while SOD2 was significantly upregulated (Figure 3A). Because antioxidant capacity was lowered in CD44‐KO cells, we performed Western blotting for GPX1, TXN, and SOD1. The protein expression of GPX1 and TXN was markedly decreased while SOD1 protein expression was not changed (Figure 3B). GPX1 is an important antioxidant enzyme reducing oxidized cellular components by converting reduced GSH to the oxidized form (GSSG).^30^ The cellular GSH content of CD44‐KO cells was significantly increased, and the ratio of GSH to GSSG was also higher than that of HuH7 cells (Figure 3C,D), suggesting the suppression of GPX1 activity in CD44‐KO cells.

### 3.4 Increased drug sensitivity of CD44‐KO cells

**Figure 4:**
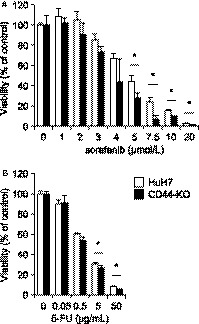
Drug sensitivity to sorafenib and 5‐FU. A, viability of cells exposed to the indicated concentrations of sorafenib for 72 h. B, viability of cells exposed to the indicated concentrations of 5‐FU for 72 h. *Open bars*, HuH7 cells; *filled bars*, CD44‐KO cells. *P < 0.05; Student's *t* test

Drug resistance is another main feature of CSCs and is acquired by the high antioxidant capacity as well as by increased expression of drug transporters.^6^ As expected, CD44‐KO cells showed an increased sensitivity to sorafenib and 5‐FU at relatively high concentrations (Figure 4), suggesting that CD44s is an important factor for drug resistance in HuH7 cells.

### 3.5 Decreased cancer stemness of CD44‐KO cells

**Figure 5:**
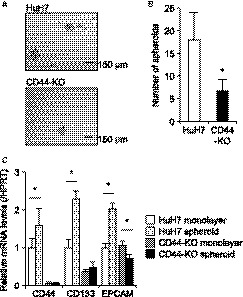
Cancer stemness. A, representative images of spheroids of HuH7 (*upper panel*) and CD44‐KO (*lower*) cells. *scale bars*, 150 µm. B, number of spheroids derived from HuH7 (*open bar*) and CD44‐KO (*filled bar*) cells. *P < 0.05, vs HuH7 cells; Student's *t* test. C, relative mRNA expression levels of CSC markers to HPRT *White bars*, HuH7 cells in monolayer culture; *light gray bars*, HuH7 spheroids; *gray bars*, CD44‐KO cells in monolayer culture; *black bars*, CD44‐KO spheroids. *P < 0.05, monolayer vs spheroids; Student's *t* test

Because CSCs have the ability of anchorage‐independent growth like normal stem cells, the spheroid culture of cancer cells has been employed to assess cancer stemness and to enrich CSCs.^31^ This assay showed a significant decrease in cancer stemness of CD44‐KO cells (Figure 5A,B). CSC markers including CD44, CD133, and EPCAM were significantly upregulated in spheroids of HuH7 cells while such upregulation was not observed in CD44‐KO cells (Figure 5C). This observation suggests that CD44s is critical for maintaining cancer stemness in HuH7 cells.

### 3.6 Involvement of NOTCH3 in CD44‐mediated maintenance of cancer stemness

**Figure 6:**
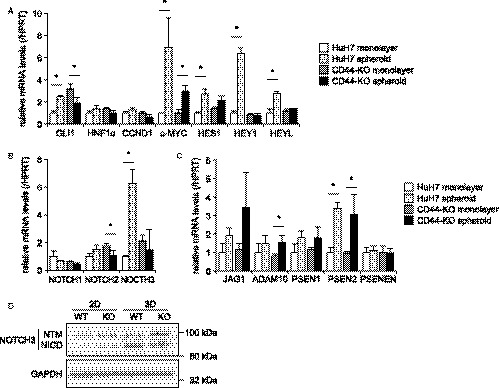
Relative mRNA expression of signaling factors to HPRT. A, hedgehog (GLI1), WNT/β‐catenin (HNF1α, CCND1, and c‐MYC), and NOTCH (HES1, HEY1, and HEYL) signaling pathways. B, NOTCH ligands. C, NOTCH signaling factors. *White bars*, HuH7 cells in monolayer culture; *light gray bars*, HuH7 spheroids; *gray bars*, CD44‐KO cells in monolayer culture; *black bars*, CD44‐KO spheroids. *P < 0.05, monolayer vs spheroids; Student's *t* test. D, Western blotting of cleaved transmembrane/intracellular fragment (NTM) and intracellular domain (NICD) of NOTCH3. GAPDH is shown for loading control

Hedgehog, WNT/β‐catenin, and NOTCH signaling pathways are well‐known and important regulatory mechanisms to maintain cancer stemness, and either of these pathways are activated in most types of CSCs.^6^ We determined the expression levels of target genes of hedgehog (GLI1), WNT/β‐catenin (HNF1α, CCND1, and c‐MYC), and NOTCH (HES1, HEY1, and HEYL) signaling pathways by RT‐qPCR. GLI1 was induced by the spheroid culture in HuH7 cells (Figure 6A). The expression of GLI1 was consistently high in CD44‐KO cells, but significantly decreased by the spheroid culture. Although HNF1α and CCND1 were not changed, c‐MYC was significantly upregulated by the spheroid culture of both cells (Figure 6A). The target genes of NOTCH signaling, such as HES1, HEY1, and HEYL, were significantly upregulated by the spheroid culture of HuH7 cells while their expression levels in CD44KO cells were not changed (Figure 6A).

We next examined the expression of signaling molecules of the NOTCH pathway. Among NOTCH receptors, only NOTCH3 showed a significant change in its expression, similar to those of the target genes (Figure 6A,B). Note that NOTCH4 expression was not detected in HuH7 cells. Other components in the NOTCH pathway were not changed (Figure 6C). The expression of NOTCH intracellular domain (NICD), which is produced by the cleavage of the NOTCH receptors, is a hallmark of the activation of the NOTCH signaling.^32^ The expression of NOTCH3 NICD in HuH7 spheroids was higher than that in CD44‐KO spheroids (Figure 6D). NOTCH1 and NOTCH2 by the spheroid culture were unlikely activated both in HuH7 and CD44‐KO cells because cleaved bands corresponding to their NICDs were not observed (Figure S3A). In addition, siRNA‐mediated knockdown of NOTCH3 in HuH7 cells resulted in a decrease in spheroid formation (Figure S3B), suggesting that NOTCH3 is an important mediator of CD44‐mediated maintenance of cancer stemness in HuH7 cells.

### 3.7 Possible existence of a CD44‐NOTCH3 axis in human HCC tissue

**Figure 7:**
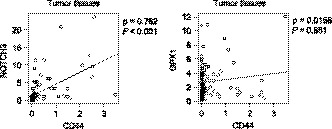
Correlation analysis of CD44 expression with NOTCH3 (*right*) and GPX1 (*left*) in HCC clinical samples. Spearman's coefficients (*ρ*) and *P* values are shown

Hepatocellular carcinoma tissues collected at Tottori University Hospital (Table 1) were used to determine the mRNA expression levels of CD44, NOTCH3, and GPX1. The expression of NOTCH3, but not GPX1, was significantly correlated with that of CD44 both in the HCC tissues (Figure 7).

## 4 DISCUSSION

Genome editing technologies including CRISPR/Cas9 have enabled us to knock‐out gene(s) in mammalian cells with high efficiency and low cost.^24^ It has been reported that NANOG‐deficient prostate cancer cells generated by the CRISPR/Cas9 system showed a significant decrease in tumorigenicity, compared with parental cells.^27^ Thus, we decided to employ this technology to study the pathological roles of CD44s in liver CSCs. However, although around 50 clones were analyzed for CD44 exon 2 sequence following the introduction of the CRISPR/Cas9 vector, we obtained only one clone that harbors the desired mutation in both alleles of the CD44 gene. Given that non‐CSCs are deficient to form a spheroid,^31^ the population of CSCs in HuH7 cells was assumed to be approximately 0.6% based on our spheroid formation assay (Figure 5B). This assumption suggests that the CD44 gene is expressed in only a small number of HuH7 cells. Because the targeting efficiency of transcriptionally inactive genes by CRISPR/Cas9 is low, additional treatment such as valproic acid may be necessary to enhance the knock‐out efficiency, in particular, of CSC marker genes.^33^

Off‐target effects are a disadvantage of the CRISPR/Cas9 system that needs to be considered, especially when knock‐out efficiency is low. Target specificity is determined mainly by a seed sequence within 12‐nt from the PAM since the introduction of a mutation into this region impaired specific cleavage by CRISPR nuclease.^25^, ^26^ Thus, we searched for potential off‐target genes by 12‐nt + PAM using an online program, by which only one gene, IKBKG was predicted (Figure S2A), possibly because the sgRNA sequence used in the present study was provided commonly by all of three online sgRNA design programs. Sequencing analysis demonstrated that no mutation was introduced into the gene, suggesting that CD44‐KO cells are valid to study the function of CD44s (Figure S2B).

Several CSC markers increase cellular antioxidant capacity.^18^, ^28^, ^29^ In colorectal carcinoma and neuroglioma cells, CD44s was reported to enhance NADPH production by activating the pentose‐phosphate pathway.^34^ This led to an increase in cellular GSH content because glutathione reductase reduces GSSG by using NADPH. CD44v reportedly also increased cellular GSH content by enhancing its biosynthesis in gastric cancer.^18^ Because CD44s lacks the domain interacting with xCT, which is required for the enhancement of GSH biosynthesis by CD44v, it has been unclear whether CD44s regulates redox status in HCC. Our data demonstrated that CD44s increases cellular antioxidant capacity by upregulating antioxidant enzymes, TXN and GPX1. The mechanism underlying how CD44s regulates GPX1 and TXN expression remains unknown. It was reported that a CD44s ligand, hyaluronic acid, upregulates and activates nuclear factor erythroid 2‐related factor 2 (NRF2) in bovine articular chondrocytes and then activated NRF2 further induces the expression of antioxidant enzymes including GPX1.^35^ The CD44s‐induced activation of NRF2 was also observed in a doxorubicin‐resistant breast cancer cell line, which possesses CSC‐like characteristics and dominantly expresses CD44s.^36^ It was demonstrated that NRF2 activation protects the breast cancer cells from oxidative stress and anticancer drugs.^36^

Among several isozymes of GPXs known in mammals, GPX1 is the most ubiquitously and abundantly expressed in most tissues, but has a limited role under a healthy condition because no obvious phenotype was observed in Gpx1 knock‐out mice.^37^ It was reported that docosahexaenoic acid (DHA) sensitized MDA‐MB‐231 breast cancer cell to doxorubicin by suppressing GPX1 activity.^38^ Of note, although oxidative stress was increased in the cells treated with doxorubicin and DHA, cellular GSH content was significantly increased.^38^ A similar observation was also made in Caco‐2 cell monolayer, where GSH content was increased by treating with mercaptosuccinate, a GPX inhibitor.^30^ In accordance with these reports, we also observed increased GSH content and GSH/GSSG ratio in CD44‐KO cells, suggesting that CD44s, unlike CD44v, enhances the consumption of GSH by activating GPX1. TXN is also another important antioxidant enzyme in normal and cancer cells.^39^ Increased oxidative stress due to the downregulation of GPX1 and TXN might contribute to sensitization of CD44‐KO cells to 5‐FU and sorafenib at least in part because both drugs increase oxidative stress in cells.^40^, ^41^

We found that GPX1 expression was significantly increased in HCC tissues (data not shown). However, CD44 makes little, if any, contribution to this upregulation at least in human HCC tissues, unlike in HuH7 cells, because no correlation between CD44 and GPX1 was observed (Figure 7). On the other hand, NOTCH3 expression was correlated with CD44 in the HCC tissues (Figure 7). In mammals, four NOTCH receptors (NOTCH1‐NOTCH4) are known, and reported to play roles in development, differentiation, and tumorigenesis.^42^ It has been demonstrated that NOTCH2 is involved in self‐renewal of CSCs in HCC.^43^, ^44^ NOTCH3 also reportedly regulates the stemness of CSCs of HCC.^45^, ^46^ Although no functional relationship between CD44s and NOTCH3 in HCC has been reported, Ma Y, *et al* observed that NOTCH3 was upregulated in nonsmall cell lung cancer tissues from chemoresistant patients and was positively associated with CD44.^47^ In addition, it was demonstrated that Her2‐negative breast tumors showed increased expression of NOTCH1 and NOTCH3.^48^ Knock‐down of NOTCH3 sensitized breast cancer cells to radiation more than did NOTCH1, and this effect was more prominent in CD44‐positive cells than in CD44‐negative cells.^48^ In the present study, it was observed that the upregulation of NOTCH3 mRNA was markedly suppressed in CD44‐KO cells while there was no significant change in the expression of NOTCH3 activators (Figure 6B,C). However, there was a discrepancy between mRNA and NICD protein expression levels of NOTCH3 in the spheroids of HuH7 and CD44‐KO cells (Figure 6B,D). These results imply that CD44 deficiency may impair the cleavage of NOTCH3 as well as its transcription. It is possible that other factors, such as ADAM17,^49^ might play a role in the activation of NOTCH3 in the downstream of CD44 in HCC cells. Although the detailed molecular mechanism remains to be clarified, our data suggest that NOTCH3 is an important mediator for the induction and maintenance of CSCs by CD44s in HCC as in other cancers.

The limitation of the present study is that we investigated the function of CD44s in one HCC cell line. However, although further studies should be carried out, the gene expression analysis of our cohort suggests that there is a close relationship between CD44 and NOTCH3 in HCC. Interestingly, although the target genes differed among cell lines, the upregulation of NOTCH3 as well as its target genes was observed in the spheroids of other HCC cell lines including HLE, HuH6, and PLC/PRF/5, whereas the NOTCH3 pathway was unlikely activated in those of HLF and HepG2 cells (Figure S3C). In addition, NOTCH3 was shown to be critical for the cancer stemness of HuH7 cells (Figure S3B). Considering these observations along with the significant upregulation of NOTCH3 in HCC tissues, NOTCH3 would be the therapeutic target of precision medicine for HCC.

In conclusion, we demonstrated that CD44s plays an important role in maintaining CSCs of HuH7 cells and regulates oxidative stress in HuH7 cells. CD44‐KO cells showed marked downregulation of NOTCH3 and GPX1, both of which are significantly increased in HCC tissues, compared with adjacent nontumor tissues. In addition, CD44 expression in HCC tissues was significantly correlated with NOTCH3 expression, suggesting that CD44 regulates CSC properties via NOTCH3. Because CD44 is expressed in normal cells as well, NOTCH3 may be a better therapeutic target for CSC‐directed therapy of CD44‐positive HCC. The clarification of molecular mechanism underlying CD44s‐induced NOTCH3 will deepen our understanding of CSCs in HCC and will provide new insights into the development and recurrence of HCC.

## CONFLICT OF INTEREST

The authors have no conflict of interest.
