# Supplementary material for: CD44 standard isoform is involved in maintenance of cancer stem cells of a hepatocellular carcinoma cell line
Source: Cancer Med. 2019 Jan 12;8(2):773–82. doi: 10.1002/cam4.1968 (PMC6382709; doi:10.1002/cam4.1968)
Supplement: Supplementary file 1 [file CAM4-8-773-s001.pdf]

Figure S1

Exon 2

WT allele …GGTCG**CTACAGCATCTCTCGGACGGAGG**CCGCT…

8 bp deletion

CD44-KO allele …GGTCG**CTACAGCA––––––––ACGGAGG**CCGCT…

…GGTCG**CTACAGC** | 256 bp insertion | **CGGAGG**CCGCT…

-CAAAGTGCTGGGATTACAGGCTTGAGCCACCGCGCCCGGCCTAATTTTTGTATTTTTAGTAGAG
ACGGGGTTTCGCCATGTTAACCAGGCTGGTCTTGAGCTCCTGACCTCAGGTGATCCACCCACC
TCGGCCTCCCAAAGTGCTGGGATTGTAGGCATGAGCCACCGCGCCCGGCCTTATTTGGCTTTT
TAAGGAGTCTGTCCTAAACTGAACTTATTACTGTCTCCAAATTATTTATGCAAAAGAATCTAACAT-
