# Supplementary material for: CD44 standard isoform is involved in maintenance of cancer stem cells of a hepatocellular carcinoma cell line
Source: Cancer Med. 2019 Jan 12;8(2):773–82. doi: 10.1002/cam4.1968 (PMC6382709; doi:10.1002/cam4.1968)

Figure S2

A

Off Target Search Query	#Off Target Sequence	Sequence	Gene	Location
20mer+PAM	0			
12mer+PAM	1	CCGCCGTCCGAGAGA	IKBKG	chrX:153775612-153775626

B

IKBKG (GRCh37/hg19:chrX:153,775,605-153,775,633)

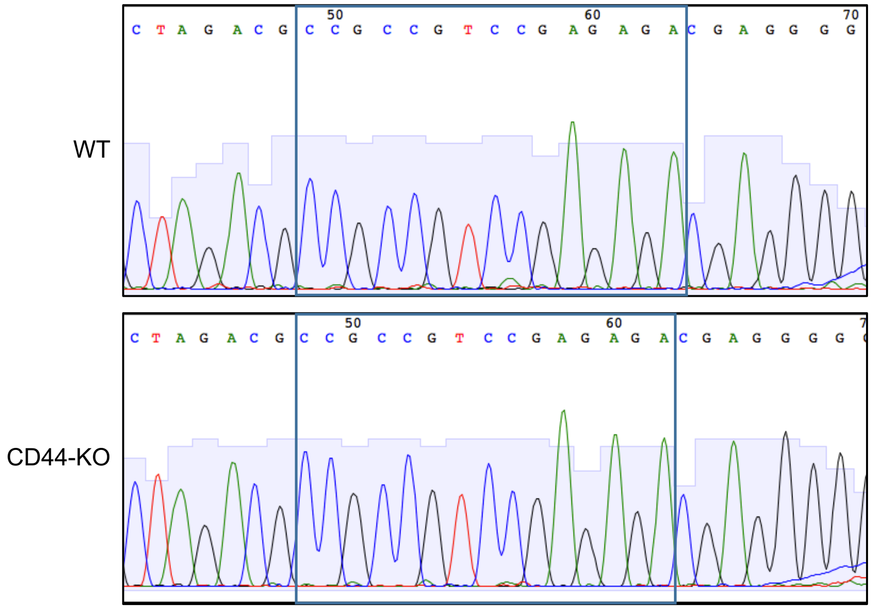

Supplement: Supplementary file 2 [file CAM4-8-773-s002.pdf]
