# Supplementary figures and images for: CD44 standard isoform is involved in maintenance of cancer stem cells of a hepatocellular carcinoma cell line
Source: Cancer Med. 2019 Jan 12;8(2):773–82. doi: 10.1002/cam4.1968 (PMC6382709; doi:10.1002/cam4.1968)

**Figure S3**

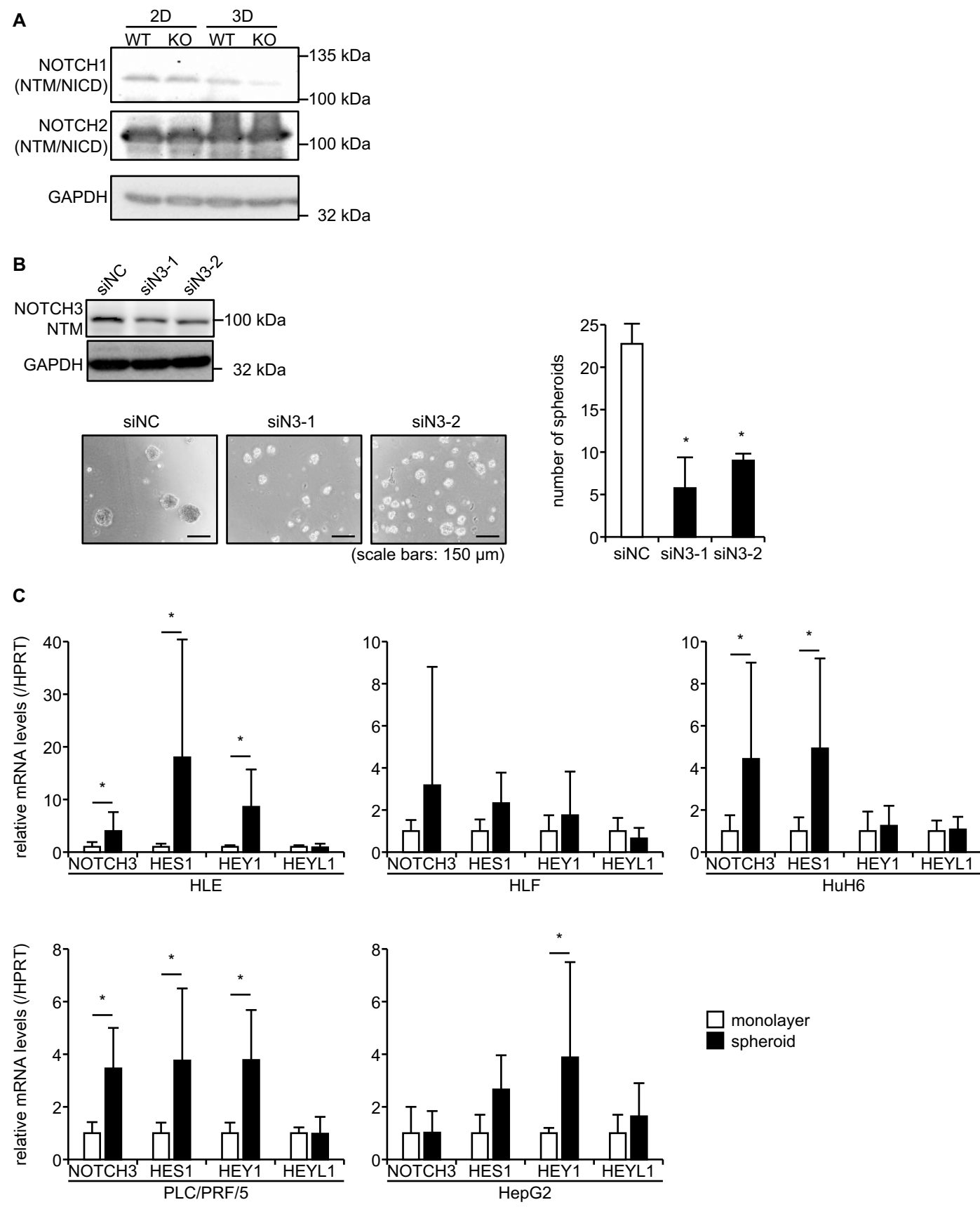

Supplement: Supplementary file 3 [file CAM4-8-773-s003.pdf]
