# Supplementary material for: CD44 standard isoform is involved in maintenance of cancer stem cells of a hepatocellular carcinoma cell line
Source: Cancer Med. 2019 Jan 12;8(2):773–82. doi: 10.1002/cam4.1968 (PMC6382709; doi:10.1002/cam4.1968)
Supplement: Supplementary file 4 [file CAM4-8-773-s004.docx]

**Table S1**

| OligoDNAs used in the present study | | |
| --- | --- | --- |
| pX458-sgCD44 construction | | |
| sgCD44 U | CACCGCTACAGCATCTCTCGGACGG | |
| sgCD44 AS | AAACCCGTCCGAGAGATGCTGTAGC | |
| sgRNA seq Rev | CGACTCGGTGCCACTTTTTC | |
| qPCR primers | sense | antisense |
| β-actin | GATGCAGAAGGAGATCACTGC | TGATCCACATCTGCTGGAAG |
| ADAM10 | ATGGGAGGTCAGTATGGGAATC | TTGGCACGCTGGTGTTTTTG |
| CAT | ACCTTTGCCTTGGAGTATTTGG | TGACCGAGAGAGAATTCCTGAG |
| CCND1 | CCCGCACGATTTCATTGAAC | AGGGCGGATTGGAAATGAAC |
| CD133 | AATTCACCAGCAACGAGTCC | AATCCATTCCCTGTGCGTTG |
| CD44 | AAGGTGGAGCAAACACAACC | TCGACTGTTGACTGCAATGC |
| EPCAM | ATAACCTGCTCTGAGCGAGTG | AACGCGTTGTGATCTCCTTC |
| GLI1 | ATGAAACTGACTGCCGTTGG | ATGTGCTCGCTGTTGATGTG |
| GPX1 | TGGCTTCTTGGACAATTGCG | AGAAGGCATACACCGACTGG |
| GPX2 | TGATGGCACCTTCCTAAACCC | TTTTTGGCCGTTTCCACACC |
| GPX3 | ATGCTGGCAAATACGTCCTC | AATGGTGCAAGCTCTTCCTG |
| GPX4 | ATACGCTGAGTGTGGTTTGC | GGCGAACTCTTTGATCTCTTCG |
| GSTZ1 | TTCGAATTGCTCTGGCCTTG | TTCAGTGCCTGGAAGTCCTTAG |
| HES1 | ACCAAAGACAGCATCTGAGC | AATGCCGCGAGCTATCTTTC |
| HEY1 | CGTGGATCACCTGAAAATGCTG | ATGCTCAGATAACGCGCAAC |
| HEYL | AAAATGCTCCATGCCACTGG | ACTCCCGAAAACCAATGCTC |
| HNF1α | ACTCCCATGAAGACGCAGAAG | TCTTGGTTGGTAGCTCATCACC |
| HPRT1 | TGGCGTCGTGATTAGTGATG | ATCTCGAGCAAGACGTTCAG |
| JAG1 | ATGACACCGTTCAACCTGAC | ATACTCAAAGTGGGCAACGC |
| c-MYC | ATGCAACCTCACAACCTTGG | TGCCCAAAGTCCAATTTGAGG |
| NOTCH1 | AATGAGTGCAACAGCAACCC | CGTTGACACAAGGGTTGGATTC |
| NOTCH2 | TGCATGCCAGGTTTCAAAGG | ACACTGCCCATTGTTCACAC |
| NOTCH3 | AGCCATGCTGATGTCAATGC | TTTTGAGCAGGGCCAAAGTG |
| PRDX1 | TGCCAAGTGATTGGTGCTTC | ACCCCATAATCCTGAGCAATGG |
| PRDX2 | TTGATGGCGCCTTCAAAGAG | TGGGGCACACAAAAGTGAAG |
| PRDX3 | AGCTTCTGATCAACGGTCCTG | TGCAGGAGTTACACGGCTAATC |
| PRDX4 | TGGGAAGGAACAGCTGTGATC | TCAAGTCTGTCGCCAAAAGC |
| PRDX5 | TTCGCTGGTGTCCATCTTTG | TGCCATCTGGTTCCACATTC |
| PRDX6 | ACTCATGGGGCATTCTCTTCTC | AATTCTGGTGCCAGCTTTGC |
| PSEN1 | AAACAAAACAGCGGCTGGTC | AAGGCTGTTGTCACTTGCAG |
| PSEN2 | ATGCTGTTTGTGCCTGTCAC | TGAATGGCGTGTAGATGAGC |
| PSENEN | AATTGAACCTGTGCCGGAAG | TTGGCTCTGTTCTGTGTAGGC |
| SOD1 | GCCACACCATCTTTGTCAGC | CTGTACCAGTGCAGGTCCTC |
| SOD2 | CTGATTGAGAATGTGGCTTCGC | AATTGGTTGCAAGGGAAGCC |
| TXN | TGGTGAAGCAGATCGAGAGC | AGGCTTGATCATTTTGCAAGG |
| TXNRD1 | ATTGCCACTGGTGAAAGACC | AAATCCAGCGCACTCCAAAG |
| TXNRD2 | AGCTTTGTTGACGAGCACAC | ATTCCAAGGCACCTTCGATG |
